# A Preliminary Study of NER and MMR Pathways Involved in Chemotherapy Response in Bladder Transitional Cell Carcinoma: Impact on progression-free survival

**DOI:** 10.22037/ijpr.2020.112646.13878

**Published:** 2020

**Authors:** Vahideh Montazeri, Mohammad Hossein Ghahremani, Hamed Montazeri, Mandana Hasanzad, d, Majid Safavi, Mohsen Ayati, Mohammad Chehrazi, Baharak Arefi Moghaddam, Seyed Nasser Ostad

**Affiliations:** a *Department of Toxicology and Pharmacology, Faculty of Pharmacy, Tehran University of Medical Sciences, Tehran, Iran. *; b *Department of Pharmaceutical Biotechnology, School of Pharmacy, International Campus, Iran University of Medical Sciences, Tehran, Iran. *; c *Medical Genomics Research Center, Tehran Medical Sciences, Islamic Azad University, Tehran, Iran. *; d *Personalized Medicine Research Center, Endocrinology and Metabolism Clinical Sciences Institute, Tehran University of Medical Sciences, Tehran, Iran. *; e *Urology Research Center, Tehran University of Medical Sciences, Tehran, Iran. *; f *Uro-Oncology Research Center, Tehran University of Medical Sciences, Tehran, Iran. *; g *Department of Biostatistics and Epidemiology, School of Public Health, Babol University of Medical Sciences, Babol, Iran. *; h *Imam Khomeini Hospital, Tehran University of Medical Sciences, Tehran, Iran. *; i *Toxicology and Poisoning Research Centre, Department of Toxicology and Pharmacology, Faculty of Pharmacy, Tehran University of Medical Sciences, Tehran, Iran.*

**Keywords:** Carcinoma, Transitional cell, Progression-free survival, Copper transporter 1 protein, Human, Cisplatin

## Abstract

One of the main genotoxic drugs used in bladder cancer chemotherapy is cisplatin. While it is applied in most types of cancers, resistance to cisplatin is wildly common. In order to overcome drug resistance, it is necessary to determine a predictive marker. This study was conducted to provide basic data for selecting and designing a gene profile for further cohort and RCT studies in the future to improve response to treatment in bladder cancer. The expression levels of ERCC1, MLH1, MSH2, and CTR1 mRNA were determined in the tumor tissue using real-time q-PCR. Progression-free survival (PFS) was analyzed in term of the level of genes expression. The results revealed that the level of ERCC1 mRNA expression was higher in the recurrence (R) group compared to the no recurrence (NR) group. Moreover, the PFS time was increased in the patients with an ERCC1 expression level of below 1.57. The level of MLH1 and MSH2 mRNA expression was lower in the R group compared to the NR group; therefore, PFS time was increased in the patients with MLH1 and MSH2 gene expression levels above the cutoff point. While the level of CTR1 mRNA expression was higher in the R group versus the NR group, the PFS time was longer in the patients with CTR1 expression levels of below 1.265 compared to the patients with high levels of CTR1 expression. It can be concluded that the level of ERCC1, MLH1, MSH2, and CTR1 mRNA expression may be associated with PFS time as possible therapeutic targets for decreasing cisplatin resistance.

## Introduction

Bladder cancer (BC) is the ninth most prevalent cancer with a global incidence of about 430,000 new cases every year. According to epidemiological studies, the highest prevalence of BC is seen in North America, Europe, and Western Asia (1, 2). 

The incidence ratio of BC in men is 3.3 time as high as in women. It is reported as the 13^th^ cause of mortality worldwide by cancer per year and tobacco smoking is known as the main risk factor (1). The Tumor- Node- Metastasis system (TNM system) is used for staging tumors, which explains tumor invasion (Tis- T_4_). Tumor grading is based on the cellular characteristics (3). Based on histological characteristics, transitional cell carcinoma (TCC) is the most common type of BC (˃90%), which originates from the uroepithelium (4, 5). There are two types of TCC with different clinical symptoms. The majority of the TCC cases (≥70%) are categorized into low-grade non-muscle invasive bladder cancer (NMIBC) subtype. NMIBC is restricted to the mucosa or submucosa and is treated locally using transurethral resection (TUR) (1, 6 and 7). Muscle-invasive bladder cancer (MIBC) is another subtype of TCC that includes 20-25% of cases and is life-threating due to metastasis and invasion to distant sites. Clinical studies reported that about 50% of the patients with this type of cancer have a poorer 5-year survival and lower quality of life (6, 7).

The recurrence and metastasis mechanism of the tumor is not clear. Therefore, knowledge of the molecular mechanisms seems to be necessary for early diagnosis and treatment decisions (8). Radical cystectomy and chemotherapy using cisplatin is the standard treatment for MIBC. However, the treatment strategy depends mostly on the clinico- pathologic pattern as well as staging (7, 9). 

Cisplatin [cisdiamminedichloroplatinum (II)], a genotoxic drug, is a platinum- based drug that binds to DNA and alters its structure. In fact, steric fluctuation in the helix by cisplatin causes higher propensity to the interstrand DNA cross-link. These lesions in the intracellular DNA structure inhibit replication and transcription of DNA, which in turn triggers apoptosis in cells (10-13). Many mechanisms contribute to drug resistance, especially resistance to cisplatin. DNA damage response (DDR) pathways play a critical role in response to chemotherapy. DDR pathways include many proteins, especially the nucleotide excision repair (NER) system that affects drug resistance through recognizing and removing DNA lesions produced by cisplatin (14, 15). In this system, Excision Repair Cross Complementing 1 (ERCC1) is the main protein and a critical factor in recognizing and removing cisplatin adducts (15, 16). 

Mismatch Repair Pathway (MMR) is another component of the DDR pathway involved in drug resistance. Mut L Homologue 1 (MLH1) and Mut S Homologue 2 (MSH2) have a critical role in the normal function of the MMR pathway (11, 17). Defects in the MMR pathway are related to cisplatin resistance, and decreased expression levels of MLH1 mRNA lead to cisplatin resistance in different cancers (18, 19). The effect of MSH2 in cisplatin resistance is also revealed in other studies (11, 20). Cisplatin is the backbone of bladder cancer treatment regimens and causes DNA damage through alkylation; therefore, faulty NER and MMR genes as components of the DNA repair pathways can cause drug resistance and tumor progression (17, 21-23).

It should also be noted that the mechanism of cisplatin resistance is multifactorial, including decreased intratumoral cisplatin accumulation due to diminished activity of transporters. Copper Transporter 1 (CTR1; SCLC31A1, or hCTR1) is the main transporter on the cell membrane contributing to cooper hemostasis with a crucial role in cisplatin uptake in tumor cells. Many studies have indicated that the level of CTR1 mRNA expression could have an impact on cisplatin resistance (10, 24-26). 

This study was conducted to evaluate association between MLH1, MSH2, ERCC1, and CTR1 expression in bladder tumor tissues and cisplatin resistance in Iranian BC patients.

## Experimental

*Patients and sample collection*

The study was done in the Urology Research Center of Sina Hospital and Uro-Oncology Research Center of Imam Khomeini Hospital affiliated of Tehran University of Medical Sciences (TUMS). It was designed according to the Declaration of Helsinki and approved by the Ethics Committee of TUMS (IR.TUMS.REC.1395.2389). The participants were recruited prospectively from April 2016 to November 2018 according to the clinicopathological diagnosis. The inclusion criteria were a histopathological report of TCC, tumor grades T2 and T3 (N0, M0), age ≤80 years old, and a negative history of treatment with radiotherapy or chemotherapy and immunotherapy. Patients who had metastatic cancer from other sites or underwent chemotherapy or radiotherapy were excluded from the study. Informed consent was taken from all participants. Fresh tumor tissues were obtained from the patients during transurethral resection (TUR) or radical cystectomy, maintained in RNA latter (Que Gene), and stored at -20 °C for RNA extraction. 

*Clinical data*

Demographic data such as age, smoking, BMI, and pathological data including tumor grading and staging were recorded. All patients received cisplatin- based chemotherapy regi-mens and response to chemotherapy was assessed four weeks after chemotherapy finished (27). All clinical documents of patients such as physical examination, imaging studies including chest X-ray and abdominal and pelvic CT scans were assessed. Response to chemotherapy was evaluated based on the criteria for solid tumor progression including recognizable tumors in other sites or in the same tissue diagnosed by cystoscopy and imaging. 

*RNA extraction and gene expression analysis using Real Time PCR*

The expression levels of ERCC1, MLH1, MSH2, and CTR1 were determined using real-time quantitative reverse transcriptase- polymerase chain reaction (real-time RT-PCR). The RNA content was isolated from the tumor tissues maintained in RNA latter (QiaGen) using Trizol reagent and stored at -70 °C. The information about the RNA yield was estimated using spectrophotometer absorbance (Eppendorf 6131, Bio Photometer) and the ratio of 260/250 nm was calculated. Complementary DNA (cDNA) synthesis was performed using a cDNA synthesis kit (Takara, Otsu, Shiga, Japan) according to the manufacturer’s instructions and stored at -70 °C. The primers are listed in Table 1. Furthermore, TBP (TATA box binding protein) and SDHA (succinate dehydrogenase complex, subunit A, flavoprotein) were determined as internal reference genes (28). The level of mRNA expression was determined with the Applied Biosystems StepOnePlus Real-time PCR using SYBR Premix Ex Taq™ II (Tli RNaseH Plus; Takara) in a 10-µL final volume. 

Data were analyzed according to the quantitative assessment of the change in the expression value of each gene relative to the housekeeping gene using 2^-(^^ΔCt^^ sample- ^^ΔCt^^ housekeeping)^. Furthermore, the expression levels of these genes were evaluated in tumors and adjacent normal samples relative to the SDHA housekeeping gene.

*Statistical Analysis*

The data are presented using mean (SD), frequency, and 95% confidence interval (95% CI). Independent sample *t*-test or Mann– Whitney U test and chi-square test were used to investigate the differences in continuous and categorical variables, respectively. The Kaplan-Meier method was applied to depict univariate survival curves illustrating the association between the biomarker expression and Progression-Free Survival (PFS). PFS was defined from four weeks after the end of chemotherapy until the time of recurrence. The log- rank test was applied to assess the statistical significance between survival curves. The Cox proportional hazard model was used to estimate the hazard ratio of recurrence for the gene expression. The cutoff point was calculated according to receiver operating characteristic (ROC) curve and Youden index. The ROC curve plotted the sensitivity (true positives) against 1- specificity (false positives), considering each value as a possible cutoff value. *p*-values less than 0.05 (*p*-value ≤ 0.05) were considered significant in all analyses. All statistical analyses were performed using the STATA version 15 (STATA Corp, College Station, Texas).

## Results

*Patients*

**Figure 1:**
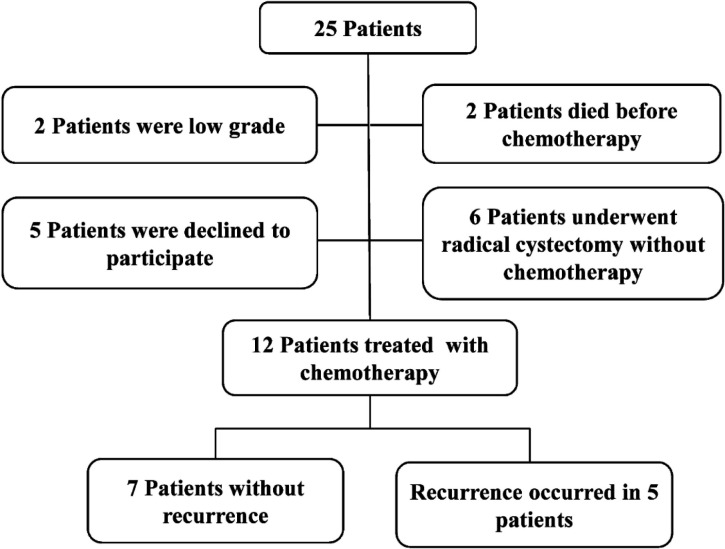
Patient's papulation

Of 25 eligible patients, 12 finished the study successfully (Figure 1). During the follow-up period, 5 patients showed recurrence on cystoscopy and were referred for radical cystectomy. There was no significant differ-ence in average age between the two groups (63 years in NR group and 62 years in R group). There was no significant difference in the smoking profile between the two groups while opium consumption was more common in the R group versus the NR group. Demographic results showed no statistically significant difference in age, smoking, opium consumption, and BMI between the two groups. Baseline characteristics of patients are summarized in Table 2. In the NR group, 25% and 75% tumor tissues were classified as T2b and T3, respectively. By contrast, in the R group, 75% and 25% of the tumors were classified as T2b and T3, respectively. There was no significant difference in the tumor grade and TMN (tumor, metastasis, node) staging between the two groups (*p*-value ≥ 0.05). 

*Assessment of gene expression in patients*

**Figure 2:**
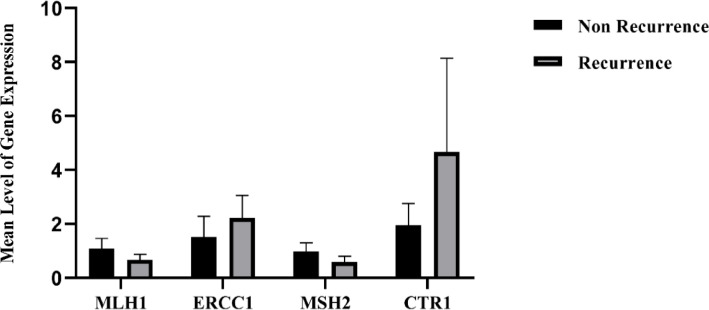
Expression levels of MLH1, ERCC1, MSH2, and CTR1 in NR and R groups (mean ± SD)

The expression levels of ERCC1, MLH1, MSH2, and CTR1 mRNA were evaluated using SYBER GREEN Real Time- PCR method. As indicated in Figure 2, the expression levels of MLH1 and MSH2 were lower in the R group compared to the NR group. By contrast, the expression levels of ERCC1 and CTR1 were higher in the R group versus the NR group. No significant relationship was observed between the mRNA expression of MLH1, MSH2, ERCC1, and CTR1 in two groups (*p*-value ≥ 0.05). 

*Level of gene expression and response to chemotherapy*

Response to chemotherapy was 58.33% in 12 patients in this study. The risk of disease recurrence was calculated using the expression levels of MLH1, ERCC1, MSH2, and CTR1 mRNA below and above the cutoff point. The risk of disease recurrence was higher when the expression levels of MLH1 and MSH2 mRNA were below the cutoff points (57.1 and 20%; *p* = 0.19, respectively). There was no correlation between the expression level of MLH1 and MSH2 mRNA and response to chemotherapy in the R group. Furthermore, the risk of disease recurrence increased when the expression levels of ERCC1 mRNA were above the cutoff point. In this group, the risk of disease recurrence when the expression levels of ERCC1 and CTR1 mRNA expression were above the cutoff point was 60% (*p *= 0.27) and 57.1% (*p* = 0.19), respectively. There was no significant correlation between the value of gene expression and tumor recurrence in the R group (Table 3). The survival of the patients in the NR group was higher when the expression levels of MLH1 and MSH2 mRNA were below the cutoff point. The survival was also increased in the NR group when the expression levels of ERCC1 and CTR1 mRNA were above the cutoff point (Table 3). No significant correlation was observed between ERCC1 and CTR1 mRNA expression levels and outcome of BC in the NR group.

*Relationship between levels of genes expression and survival of patients*

As shown in Table 4, the median survival was calculated for each gene above and below the cutoff point values. Based on the results, patients with expression levels of MLH1 and MSH2 below the cutoff point had a median survival of 17 months (95% CI = 0-45.22) and 6 months (95% CI = 4.17-7.28), respectively. It was clearly demonstrated that the patients with MLH1 mRNA expression level below the cutoff point were high risk patients compared to patients with high levels (HR: 2.64, 95% CI = 0.27-25.56). Additionally, the PFS of patients with MSH2 mRNA expression level below the cutoff point was better compared to patients with expression levels above the cutoff point (HR:0.27, 95% CI = 0.03-2.47) (Table 4). On the contrary, the median survival of patients with expression levels of ERCC1 and CTR1 mRNA above the cutoff point was 6 months (95% CI = 3.85-8.14) and 6 months (95% CI = 0.2-33.79), respectively. Interestingly, poorer PFS was noted in patients with expression levels of ERCC1 and CTR1 above the cutoff point (HR: 4.6, 95% CI = 0.47-44.33 and HR: 3.08, 95% CI = 0.34-27.75, respectively) compared to patients whose expression levels were below the cutoff point (Table 4). 

**Figure 3.**
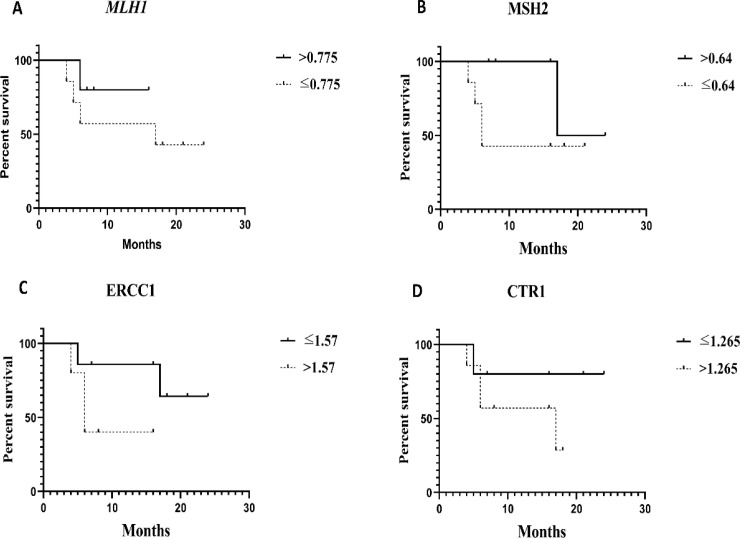
Evaluation of recurrence in patients with gene expression levels below and above the cutoff point. (A and B) Patients with MLH1, MSH2 mRNA expression levels above the cutoff point had longer the PFS compared to other patients. (C and D), Patients with ERCC1 and CTR1 mRNA expression levels above the cutoff point had shorter the PFS compared to other patients

The PFS rates were assessed for expression levels of MLH1, ERCC1, MSH2, and CTR1 mRNA schemed by Kaplan-Meier survival curves (Figure 3). The results showed any significant difference between R and NR groups. It seems that increasing the sample size will produce significant differences in the results of other studies.

**Table 1:** List of primer

Gene		Primer
**ERCC1**	Forward Reverse	TTTGGCGACGTAATTCCCGAC
		CCTGCTGGGGATCTTTCACA
**MLH1**	Forward Reverse	CTCTTCATCAACCATCGTCTGG
		GCAAATAGGCTGCATACACTGTT
**MSH2**	Forward Reverse	AGTCAGAGCCCTTAACCTTTTTC
		GAGAGGCTGCTTAATCCACTG
**CTR1**	Forward Reverse	GGGGATGAGCTATATGGACTCC
		TCACCAAACCGGAAAACAGTAG
**SDHA**	Forward Reverse	CGGGTCCATCCATCGCATAA GACGTGCAGCTGAAGTAGGT
**TBP**	Forward Reverse	CAGCTTCGGAGAGTTCTGGG TATATTCGGCGTTTCGGGCA

**Table 2:** Demographic and clinicopathological characteristic of patients

Characteristics	NR (n = 7)	R (n = 5)	*p*-value
**Age**	63 ± 10.94	62.40 ± 5.68	0.64
**BMI**	27.22 ± 3 .92	27.60 ± 7.46	0.75
**Smoking, (%)** **No** **yes **	2 (100) 5 (50)	0 (0) 5 (50)	0.47
**Opium, (%)** **No** **yes**	6 (66.7) 1 (33.3)	3 (33.3) 2 (66.7)	0.52
**Grade, (%)** **T2b** **T3**	1 (25) 6 (75)	3 (75) 2 (25)	0.22
**Stage, (%)** **2** **3**	1 (25) 6 (75)	3 (75) 2 (25)	0.22

NR: No recurrence group; R: Recurrence group.

**Table 3.** Assessment of relationship between risk of recurrence and gene expression levels below and above the cutoff point

Gene Cutoff point	NR, n (%)	R, n (%)	*p*-value
**MLH1** **>0.775** **≤0.775**	4 (80) 3 (42.9)	1 (20) 4 (57.1)	0.19
**ERCC** **≤1.57** **>1.57**	5 (71.4) 2 (40)	2 (28.6) 3 (60)	0.27
**MSH2** **>0.64** **≤0.64**	4 (80) 3 (42.9)	1 (20) 4 (57.1)	0.19
**CTR1** **≤1.265** **>1.265**	4 (80) 3 (42.9)	1 (20) 4 (57.1)	0.19

NR: no recurrence group; R: recurrence group.

**Table 4:** Assessment of relationship between survival and gene mRNA expression levels

Gene Cutoff point	Number of events	Median Survival Month (95% CI)	Hazard Ratio (95% CI)	*p*‐value^a^
**MLH1** **≤0.775** **>0.775**	4 1	17 (0-45.22) .	2.64 (0.27-25.56)	0.4
**ERCC** **≤1.57** **>1.57**	2 3	. 6 (3.85-8.14)	4.60 (0.47-44.33)	0.18
**MSH2** **≤0.64** **>0.64**	4 1	6 (4.71-7.28) .	0.27 (0.03-2.47)	0.25
**CTR1** **≤1.265** **>1.265**	1 4	. 17 (0.2-33.79)	3.08 (0.34-27.75)	0.31

^a^Log-Rank *p*-value.

## Discussion

One of the main challenges of clinicians is drug resistance and response to chemotherapy agents. The optimal effect of chemotherapy agents on tumor cells can be achieved by clarifying their molecular biology, which results in reduced resistance and increased patient survival. Considering the complexity of chemotherapy response, predicting the outcome of chemotherapy agents can be beneficial for treatment decision-making. Therefore, a unique therapeutic regime is necessary to overcome drug resistance. Identification of mechanisms of response to anticancer drugs and the genetic profile of the patients would make it possible to use personalized drugs based on molecular patterns. Anticipation of response to chemotherapy can be helpful for clinical decision-making to find optimal treatment strategies. Cisplatin resistance is widely common in patients treated with cisplatin-based chemotherapy (29). Several molecular mechanisms are involved in cisplatin resistance in different cancers. Among these mechanisms, NER and MMR pathways not only have a critical role in genetic stability, but are also involved in drug resistance and response to chemotherapy (18, 30-32).

No molecular biomarkers have yet been identified to overcome drug resistance in Iranian BC patients. Therefore, ERCC1, MLH1, MSH2, and CTR1 genes were selected in order to suggest a genetic panel for Iranian BC patients as a promising approach for clinical trials or cohort studies in the future. 

Studies have demonstrated that low expression of ERCC1 within the tumors improves the response to chemotherapy based on cisplatin and the outcome of cancer by decreasing the removal of cisplatin- induced DNA adducts resulting in increased survival (21, 33-35). Mullane *et al.* reported that DNA repair pathway largely contributed to prognosis of MIBC patients. The results of the expression level by immunohistochemistry showed that high expression levels of ERCC1 played a key role in survival of patients who received cisplatin as the first line of chemotherapy (35). The results demonstrated that ERCC1 mRNA expression levels above the cutoff point might lead to decreased survival in patients. Moreover, the expression level of ERCC1 was higher in the R group compared to the NR group although the difference was not statistically significant, which can be explained by the small sample size of the study. 

Defects in genetic integrity may lead to disorders in the MMR system, especially MSH2 and MLH1. Consequently, defects in these factors alter the propensity of invasive tumor cells for metastasis and change their sensitivity to chemotherapeutic drugs (18, 22). Several studies revealed that lack of MLH1 and MSH2 could induce cisplatin resistance in tumor cells, whereas low expression levels of MLH1 and MSH2 resulted in poor survival in ovarian and bladder cancer patients treated whit platinum drugs (36-38). In a study by Goodspeed *et al*., MSH2 knockdown in bladder cancer cell lines resulted in apoptosis- induced cisplatin reduction. Furthermore, the results demonstrated decreased survival in MIBC with a low expression level of MSH2 protein after treatment with platinum- based chemotherapy (37). 

We investigated whether MLH1 and MSH2 mRNA expression levels below the cutoff point had any effects on the poor survival of these patients but the results were not significant because of the small sample size. Generally, this pattern was observed in both groups of patients, which can be used for designing RCT and cohort studies in the future. Moreover, it should be noted that the expression of these genes was reduced in the R group compared to the NR group. However, the results may vary according to the cancer type, for instance, some studies showed that low levels of MSH2 expression were associated with a high survival in non- small cell lung cancer and were not associated with a poor survival in ovarian cancer (39, 40). 

Other mechanisms that attribute to cisplatin resistance include decreased accumulation of cisplatin due to low expression of transporters. Among them, studies have shown that CTR1 regulates the copper intracellular concentration, which plays a crucial role in the uptake or influx of cisplatin. Therefore, low levels of CTR1 mRNA expression reduce cisplatin sensitivity (25, 41 and 42). Kilari *et al.* investigated the correlation between CTR1 expression and pathological outcome in pre- and post- chemotherapy tissues and suggested that CTR1 immuno-expression could be used as a biomarker before NAC (neoadjuvant chemotherapy)- TURBT in order to predict the risk of NAC for individual patients (25). To the best of our knowledge, the correlation between CTR1 and survival of BC patient is not clear. In our study, the CTR1 expression level was higher in the R group compared to the NR group, which was associated with increased survival. In addition, our results failed to obtain statistical significance that can be explained by the small sample size. 

One of the limitations that can be highlighted is the exclusion of patients who underwent other treatment strategies. Other limitations were difficulty in obtaining fresh tumor tissues during the surgery and follow-up patients after chemotherapy. Another limitation was conducting the study in two centers while more centers were needed, which was not allowed. In addition, this report is related to a small sample of Iranian patients and is indeed a primary study for selecting the genes that can be used as markers in future investigations. Moreover, longer follow-up times are required to prevent bias. We could not unravel the relationship between the expression level of these genes as molecular markers and response to chemotherapy. However, in these patients, response to chemotherapy during the follow-up period was consistent with our hypothesis. 

## Conclusion

In conclusion, the expression levels of ERCC1, MLH1, MSH2, and CTR1 were assessed in BC patients. The PFS and risk of disease recurrence were successfully measured. Despite a non-significant difference in gene expression between R and NR groups, a correlation was observed between gene expression level and risk of disease recurrence that was consistent with other studies. Therefore, these genes can be suggested as a model of gene profiling for personalized cancer treatment to not only reduce treatment costs but also to increase the survival of the patients. The suggested model of gene profiling can be a promising strategy for cohort and clinical studies with larger sample sizes in different populations. 
